# Panmim: a resource of pan-cancer metastasis immune microenvironment

**DOI:** 10.1186/s12967-025-06484-5

**Published:** 2025-10-28

**Authors:** Xuelu Zhang, Simeng Hu, Huanjing Hu, Yongkun Li, Pengyin Nie, Xichen Hu, Ruidong Xue, Xiao Xiang, Lu Zheng

**Affiliations:** 1 Department of Hepatobiliary Surgery, The Second Affiliated Hospital, Third Military Medical University (Army Medical University), Chongqing, 400037 China; 2 Precision Diagnosis and Treatment Laboratory for Hepatobiliary Diseases, The Second Affiliated Hospital, Third Military Medical University (Army Medical University), Chongqing, 400037 China; 3 Peking University-Yunnan Baiyao International Medical Research Center, International Cancer Institute, Department of Medical Bioinformatics, School of Basic Medical Sciences, Peking University Health Science Center, Beijing, China; 4 Translational Cancer Research Center, Peking University First Hospital, Beijing, China

**Keywords:** Cancer metastasis, Immune microenvironment, ScRNA-seq, Gene expression, Database

## Abstract

**Background:**

Cancer metastasis, the process by which tumor cells spread from the primary site to other organs, is one of the leading causes of death in cancer patients. However, due to its complexity and unpredictability, the study of cancer metastasis remains a significant challenge in medicine. In recent years, with the advancement of high-throughput sequencing technologies and single-cell transcriptomics, we could gain a deeper understanding of the molecular mechanisms and cellular heterogeneity underlying cancer metastasis. There is an accumulating volume of publicly available cancer metastasis research data. Nevertheless, these resources lack proper organization, hindering systematic analysis.

**Methods:**

In this study, we developed an integrated resource named Panmim (http://www.gdwk-bioinfo.com/pan_metastasis/home) to investigate the immune microenvironment of metastatic tumors. The database currently encompasses 90 single-cell RNA-seq datasets from metastatic cancers of diverse origins, encapsulating 14 distinct metastatic sites and 36 primary cancer types. Panmim facilitates a cellular-level comparison of similarities and differences between primary and metastatic cancers, encompassing pathway analysis, alterations in cellular metabolic pathways, cellular distribution preferences, and various aspects of intercellular communication.

**Results:**

Panmim presents the analysis results through an intuitive interactive graphical interface, enabling users to explore and understand the complex biological phenomena during cancer metastasis more conveniently. This resource will become a valuable tool for biologists and bioinformaticians to study the mechanisms of cancer metastasis, providing important data support and scientific basis for the optimization of cancer treatment strategies.

**Supplementary Information:**

The online version contains supplementary material available at 10.1186/s12967-025-06484-5.

## Introduction

Metastasis is a critical process in cancer progression, where malignant cells detach from the primary tumor site and travel through the bloodstream or lymphatic system to colonize distant organs. This phenomenon is a major cause of mortality in cancer patients, accounting for the vast majority of cancer-related deaths [1, 2]. However, it is still not well understood. During the complex process of cancer metastasis, a plethora of elements are instrumental in facilitating this multifaceted event. These elements include, but are not limited to, the Epithelial-Mesenchymal Transition (EMT) [3], an array of genomic mutations [4], significant alterations in cellular metabolic traits [5], the remodeling of the extracellular matrix [6], and intricate interactions within the tumor’s immune microenvironment [7]. Indeed, the invasion of adjacent tissues and the seeding of distant sites to form metastases remain core characteristics of malignant cancers. Understanding the dynamics of this process will aid in identifying potential targets for molecular therapies that could halt or potentially reverse the growth and spread of cancer.

Aberrant gene expression plays a pivotal role in the process of cancer metastasis [8]. In recent years, the advancement and maturation of high-throughput sequencing technologies have generated an abundance of sequencing data, offering invaluable resources for cancer metastasis research [9, 10]. Among these, RNA-seq has become an essential tool in transcriptomics, enabling comprehensive gene expression profiling that delves into the biological characteristics of cells and tissues [11]. However, conventional RNA-seq methods face limitations when studying tissues or cell populations, as they fail to expose intercellular heterogeneity. The advent of single-cell RNA sequencing (scRNA-seq) has addressed this challenge. scRNA-seq reveals genetic expression heterogeneity at the single-cell level, significantly enriching our understanding of cell types, differentiation pathways, and functional states. Multiple tumor single-cell databases have also been established, such as TISCH [12], SC2 disease [13] and CancerSCEM [14]. These databases collect a large number of single-cell transcriptomic sequencing datasets related to cancer and process them according to a unified analysis workflow. In addition, online platforms for analyzing and visualizing single-cell data as well as spatial transcriptome data, such as STExplore [15], have also been developed. These sites are widely used in cancer research. This advance has also pioneered a new perspective in cancer metastasis research [16, 17]. With the widespread application of single cell sequencing technology, we have the opportunity to gain a deeper understanding of the molecular mechanisms and cellular heterogeneity underlying cancer metastasis. There is an accumulating volume of publicly available cancer metastasis research scRNA-seq data [18–20].

Currently, the majority of single-cell sequencing data is stored in public databases such as GEO [21], ArrayExpress [22], and NGDC [23] in their raw form. However, this format does not provide users with direct analytical outcomes. Several databases related to cancer metastasis have recently been developed to display gene expression and functional information. For instance, CMGene [24] is a database dedicated to the collection of cancer metastasis-related genes through literature search, without involving omics data analysis. LncR2 metasta [25] focuses more on the relationship between long non-coding RNAs and cancer metastasis. Currently, there are two published omics databases related to tumor metastasis, HCMDB [26] and metsDB [27]. Despite their usefulness, they cover limited types of primary cancers and lack focus on single-cell metabolic levels. In addition, HCMDB has currently stopped access.

In this study, we introduce a database named Panmim. Panmim is a database to parse the immune microenvironment of metastatic cancer and accessible without the need for login and is fully operational at http://www.gdwk-bioinfo.com/pan_metastasis/home. Users can freely access it. Panmim provides 90 scRNA-seq datasets with 3,947,298 single-cell transcriptomics. These datasets encompass 36 different types of cancer across 14 distinct metastatic sites (e.g. liver, brain). As the most comprehensive single-cell transcriptomics-based cancer metastasis database, Panmim will be a valuable resource for bioscientists and bioinformaticians.

## Materials and methods

### Data collection and management

All single-cell sequencing datasets and metadata in the Panmim were sourced from the GEO, ArrayExpress, and NGDC databases. On the search page, ‘metastasis’, ‘cancer’ and ‘scRNA-seq’ are used as keywords for retrieval. Datasets were manually screened with exclusion criteria including: Datasets containing only primary cancer samples and normal samples; Not single-cell transcriptome sequencing data; Non-tumor metastasis data. Datasets containing single-cell transcriptome sequencing datasets with tumor metastases or both metastases and primary samples were retained. A total of 90 datasets were finally collected. In the data set management section, we will organize the data according to the principle that each data set contains only one tumor metastasis site. If a dataset contains multiple metastases, we split it into multiple independent datasets based on those metastases. After a unified process of quality control, removal of doublets, and deletion of low-quality cells, 3,947,298 cells were retained. Among them, there are 232 primary cancer samples, 336 metastatic cancer samples, and 59 normal samples.

### scRNA-seq data analysis

All single-cell sequencing data analysis was conducted using Seurat (v4.4.0) [28]. The quality control process is as follows. First, we calculated the mitochondrial content in each cell and then determined a threshold, which represents 60% of the maximum mitochondrial content among all cells. Cells above this threshold were considered dead and removed. In addition, nFeature_RNA and nCount_RNA also use 70% of the maximum value across all cells as thresholds. Ultimately, we filter out cells that meet the following criteria for subsequent analysis: nFeature_RNA is greater than 250 and less than 70% of its maximum value, nCount_RNA is less than 70% of its maximum value, and mitochondrial content is less than 60% of its maximum value. Subsequently, the R package DoubletFinder (v2.0.4) [29] was used to remove doublet cells. Harmony [30] was used to eliminate batch effects between samples. Next, we normalize the data using the NormalizeData function and identify highly variable features using FindVariableFeatures. FindAllMarkers is then used to identify marker genes for various clusters. During the cell annotation process, to comprehensively annotate cells from different tissue types, we manually collect classic marker genes as well as cell annotation labels used in the original articles of each dataset. Meanwhile, we integrate marker genes from CellMarker 2.0 [31] and the reference gene set from scType [32] to determine the final set of genes for cell annotation. Finally, we complete the annotation of each cluster using the sctype_score function from the scType package.

### Differential expression analysis and enrichment analysis

For datasets containing biological replicates, we employed the DESeq2 [33] method for differential expression analysis. For datasets without biological replicates, the edgeR [34] method was utilized. Through these analyses, we aim to identify differentially expressed genes between different sample groups (e.g. primary tumors versus metastatic tumors). Subsequently, we utilized the R package clusterProfiler [35] to conduct pathway enrichment analysis on the identified differentially expressed gene sets to elucidate their functions.

### Metabolic pathway activity analysis

The analysis of metabolic pathway activity utilized the R package scMetabolism [36], which is designed to quantify metabolic activity at single-cell resolution. sc.metabolism.Seurat function is used to calculate the metabolic pathway scores for the cells. We select the most abundant KEGG type as the metabolism.type. Then, we calculate the average metabolic pathway scores for each group and cell type, which will be used as the final scores for visualization display.

### Tissue distribution of clusters

To analyze the tissue distribution preferences of each cellular subpopulation, we calculated Ro/e scores [37]. Ro/e is the ratio of observed cell number over the expected cell number of each cell cluster and tissue, in which the expected cell number for each cell cluster and tissue are obtained from the chi-squared test.

### Cell–cell community

Cell communication refers to the process of information exchange between cells through various signaling molecules, which plays a crucial role in cancer metastasis. We calculated the communication situation and ligand-receptor pairs among various cell subpopulations using the R package CellChat [38]. Expression matrix and meta data of the samples are extracted from Seurat, and then the CellChat object is generated by createCellChat function. The computeCommunProb function is used to infer the communication probability between cells. Subsequently, the subsetCommunication function is used to extract communication information between specific cell populations.

### Database construction

The online database framework is built with Django, and deployed on a centOS environment with NGINX and uWSGI. The front end is developed using HTML, JavaScript, jQuery, and CSS. Data visualization is achieved using Plotly, Echarts, and Highcharts. All tables on the website are built using datatables. In terms of data storage, the gene expression data of each dataset is saved in pickle format for quick and easy reading; other files are saved in txt or csv format. The data on the download page is stored in RData format.

## Results

### Overview of database

Panmim contains 90 single-cell sequencing datasets, encompassing 14 different metastatic sites from 36 distinct types of primary cancer (Additional file 1: Table S1). The most representative disease types in these datasets include breast cancer, colorectal cancer, and head and neck squamous cell carcinoma. The most studied metastasis sites mainly include the brain, liver, and lymph nodes. There are a total of 627 samples in Panmim, which include 232 primary cancer samples, 336 metastatic cancer samples, and 59 normal samples. We classified all datasets. If a dataset contains both primary cancer samples and metastatic samples, we categorize it as a ‘Comparison’. If a dataset only contains metastatic samples or contains both metastatic samples and normal samples, we categorize it as ‘Single’. According to the statistics, there are 52 datasets in the Comparison dataset and 38 datasets in the Single dataset. After quality control, a total of 3,947,298 cells were retained. The above information is displayed interactively on the statistics page.

### Website interface

**Fig. 1:**
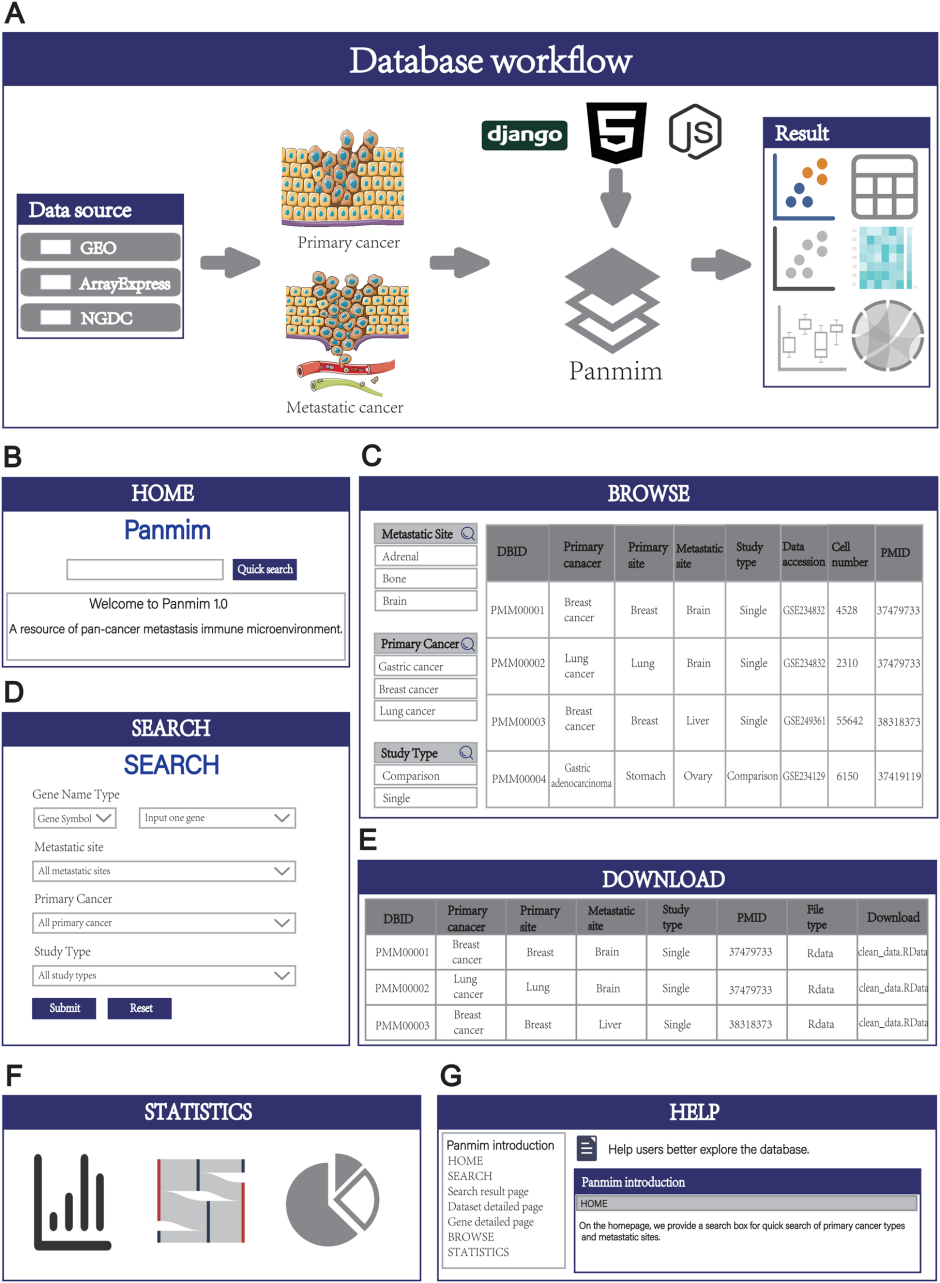
Workflow and contents of Panmim. **A** Overall design and workflow of database. **B** ‘HOME’ page, users can quickly search by keyword. **C** The ‘BROWSE’ page shows all the datasets contained in this database. The filter box is provided on the right to filter the data. **D** On the ‘SEARCH’ page, users are able to narrow down the desired data set by utilizing a series of filters. **E** ‘DOWNLOAD’ page, which allows users to download Seurat objects for each dataset. **F** The ‘STATISTICS’ page interactively displays the number of datasets and cell counts contained for each primary cancer type and metastatic site. **G** The ‘HELP’ page offers a comprehensive user guide to assist users in quickly getting started with using Panmim

The workflow of Panmim is presented in Fig. 1A. The Panmim database provides six user-friendly functional pages, including ‘HOME’, ‘BROWSE’, ‘SEARCH’, ‘DOWNLOAD’, ‘STATISTICS’, and ‘HELP’. The ‘HOME’ page provides a succinct overview of the fundamental features and capabilities within the database (Fig. 1B). First of all, there is a search box, through which users can quickly retrieve the datasets that they are interested in. Secondly, there is a detailed introduction to the website so that users can quickly master the relevant information about the website. Next, there is a statistics of the datasets contained in the website, and a word cloud is used to display all the primary cancer types. Subsequently listed is all the information about the metastasis sites involved in the website. Finally, there is a news section on the website, which enables users to keep abreast of the updating dynamics of the website in a timely manner. On the ‘BROWSE’ page, users can intuitively access all datasets included in this database and their related information (Fig. 1C). The ‘SEARCH’ page is designed to help users quickly locate datasets related to cancer types or metastatic sites of interest using precise filtering conditions (Fig. 1D). The ‘DOWNLOAD’ page provides users with the Seurat data for each single-cell dataset, saved in RData format, which users can freely download (Fig. 1E). The ‘STATISTICS’ page is used to display statistical results of the data in the database (Fig. 1F). If users need assistance, the information provided on the ‘HELP’ page can effectively aid them in better exploring Panmim (Fig. 1G).

### Data retrieval

In Panmim, we offer two main data retrieval methods: Quick Search and Advanced Search. Quick Search is located on the ‘HOME’ page and offers a rapid and streamlined method for data retrieval (Fig. 1B). When using it, users simply input keywords such as cancer type (Breast cancer) or metastatic site (Brain), and datasets containing these keywords will be displayed on the ‘SEARCH RESULT’ page. The Advanced Search feature is located on the ‘SEARCH’ page and offers users a more precise search experience (Fig. 1D). Users can first look up genes of interest by searching with the gene symbol or Entrez ID. Then, users can select the primary cancer type or cancer metastatic site of the study. Finally, users can choose the study type, including two options: Comparison and Single. Clicking Submit will retrieve the desired datasets. Additionally, on the ‘HOME’ page, we have displayed all primary cancer types in a word cloud chart format. Users can click on any cancer type to perform a search for that specific type.

### Resource exploration and browsing

On the ‘BROWSE’ page, users can browse and download all the datasets and related information contained in the database, including primary cancer type, primary tissue, metastatic site, study type, data source, cell count, and references. To facilitate users in finding specific datasets, we have set up a data filtering function on the left side of the web page, allowing users to quickly locate the required datasets based on filtering criteria.

### Dataset details

**Fig. 2:**
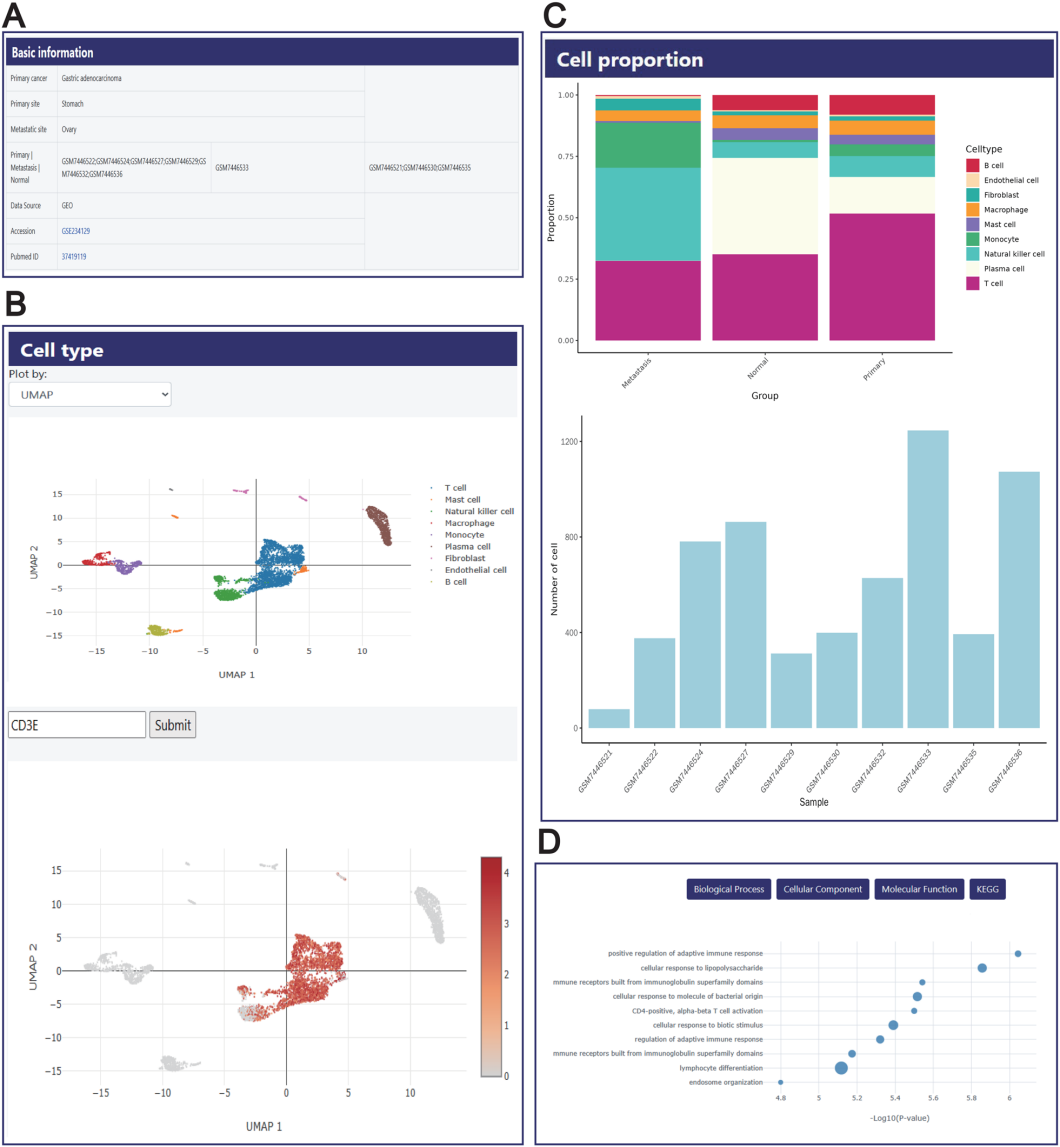
Details page content for the dataset. **A** This section presents the basic information of the dataset, including metadata, sample grouping, data sources, and references. **B** This part presents the cell annotation results of the dataset in the form of UMAP and t-SNE and provides the function of gene expression query. **C** Cell proportion shows the proportion of cell population in each sample and the number of cells in each sample. **D** The figure shows the enrichment results of GO and KEGG of differential genes to explore their biological functions

**Fig. 3:**
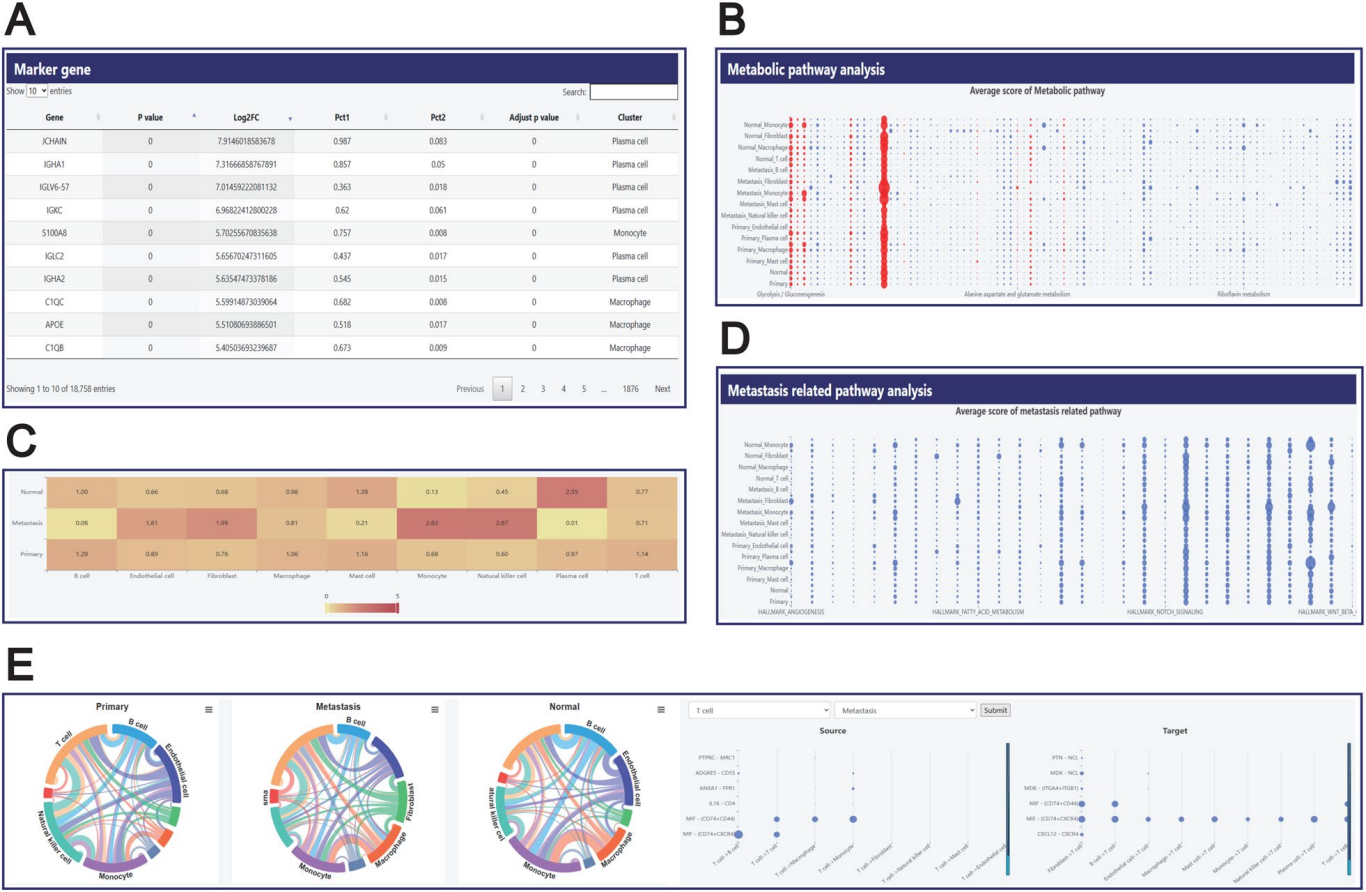
Details page content for the dataset. **A** Marker gene for each cell subpopulation. **B** The scoring of each metabolic pathway in different cell subsets. **C** Ro/e scores of each cell group in different groups. **D** Tumor metastasis-related pathway scores of different cell subsets. **E** This diagram shows the crosstalk between different cells

In the ‘SEARCH RESULT’ page or ‘BROWSE’ page, by clicking the DBID of each dataset, users can be redirected to the detailed information page of that dataset. This page provides comprehensive information related to the dataset, mainly including two parts: the first part includes basic information about the dataset, such as sample grouping, data sources and references (Fig. 2A). It is convenient for users to quickly understand the datasets and master the key information; the second part displays the analysis results of the dataset. Firstly, in the ‘Cell type’ section, users can intuitively observe the cell clustering and types within the dataset through two visualization methods: UMAP(Uniform Manifold Approximation and Projection) and t-SNE(t-distributed Stochastic Neighbor Embedding). Additionally, we have added a search box on the right side of the interface to facilitate users in querying gene expression levels across various cell types (Fig. 2B). In the ‘Cell proportion’ section, we show the proportion of cell types in each group and the total number of cells in each sample using stacked bar charts and histograms respectively (Fig. 2C). To explore the differences in biological functions between primary and metastatic cancer, we conducted functional enrichment analysis on the differentially expressed genes between these two groups, including GO and KEGG pathway analysis (Fig. 2D). This analysis helps reveal the differences in gene expression patterns under the two cancer states, thereby aiding users in better understanding the potential molecular mechanisms of metastasis. These analytical results are only presented in the Comparison sample set. Additionally, we also provided the marker genes for each cell type in a tabular format (Fig. 3A). The table supports sorting and filtering by users in multiple dimensions such as gene name, fold change, p value. Metabolism plays a important role in the process of cancer metastasis, and metastatic tumors exhibit different metabolic characteristics compared to primary tumors [39]. Therefore, in the Metabolic pathway analysis section, we evaluated the scoring of 85 metabolic pathways in different groups and cell types, and at the same time, through literature review, sorted out 10 metabolic pathways related to tumor metastasis, and highlighted the corresponding scores of these pathways in red on the website. (Fig. 3B). Here, users can view the metabolic score profile of each group under each metabolic pathway, and also delve into the score differences of different cell types. Subsequently, we calculated the Ro/e scores for each cell type, which indicates the ratio of observed cells to expected cells (Fig. 3C). This is an indicator used to quantify the preference degree of each cell subgroup for tissues, and it is commonly used to assess the distribution preference of specific cell subgroups under different tissues or conditions. This section is presented in the form of an interactive heatmap and is only available within the comparison dataset. In addition, by reading the literature, we screened out 28 pathways related to tumor metastasis and calculated the scores of these pathways in different groups and cell types (Fig. 3D). Lastly, we calculated the crosstalk between different cell types (Fig. 3E). To this end, we used chord diagrams to display the total communication strength between cells. Additionally, we conducted a detailed analysis of the specific ligand-receptor pairs involved in communication between different cell types acting as sources and targets in various groups.

**Fig. 4:**
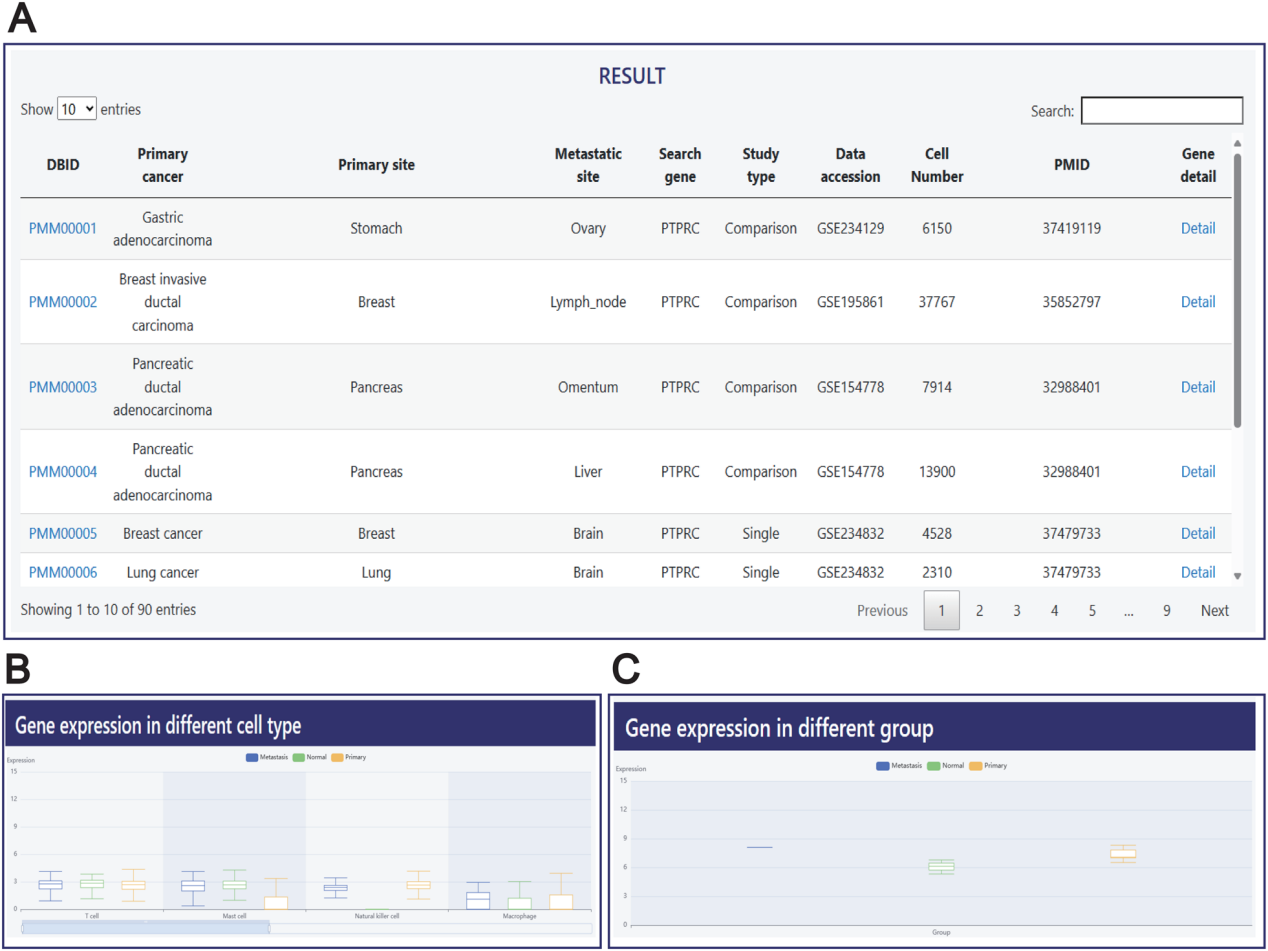
Gene details page. **A** Search results page for genes. **B** This figure shows the expression of genes in different cell types. **C** This section displays the expression of genes within different groups

When users search for genes through the ‘SEARCH’ page, two additional columns will be added to the ‘SEARCH RESULT’ page (Fig. 4A). One column displays the gene being searched for, and the other contains hyperlinks. Users can click on the ‘Detail’ button in the last column to be redirected to the detailed page of the gene. The gene detail page is composed of two parts. The first part, named ‘Gene expression in different cell type’, primarily displays the expression of the gene across various cell types (Fig. 4B). The second part, named ‘Gene expression in different groups’, focuses on showcasing the gene's expression across different groupings (Fig. 4C).

### Case study

Next, we will take a specific case for systematic analysis to demonstrate the value of Panmim in promoting research related to cancer metastasis. Enter the search page, select ‘Ovary’ in the Metastatic site tab, then choose ‘Gastric adenocarcinoma’ in the Primary Cancer tab, click ‘Submit’, and find a related dataset (PMM00001). Then click on the DBID to enter the details page. This dataset detected a total of 6,150 cells and identified 9 types of cells, including T cells, B cells, Mast cells, Natural killer cells, Macrophage, Monocytes, Plasma cells, Fibroblast, and Endothelial cells (Fig. 2B). By analyzing the cell proportions, we found that compared to the primary group, the percentage of fibroblasts in the metastatic group is higher. According to relevant literature reports, fibroblasts play an important role in tumor metastasis [40]. Moreover, fibroblasts in the metastatic group have a higher score in angiogenesis pathways. Cell communication analysis shows that there is more interaction between fibroblasts and endothelial cells in the metastatic group. Guo et al. found that the mCAF1 subpopulation promotes lymph node metastasis of esophageal squamous cell carcinoma (ESCC) by promoting angiogenesis [41]. Based on these research results, we speculate that fibroblasts interact with endothelial cells to promote angiogenesis, thereby driving the occurrence of metastasis. In addition, through enrichment analysis, we found that in the metastatic samples, more pathways related to inflammatory responses were altered, such as the Toll-like receptor signaling pathway, TNF signaling pathway, regulation of adaptive immune response, and lymphocyte differentiation. These pathways play important roles in the process of cancer metastasis [42]. The Ro/e results indicate that NK cells are highly enriched in the metastatic group, these cells highly express some genes related to cytotoxicity, such as GNLY, NKG7, GZMB, KLRD1, and PRF1. Based on the results of cell communication, we found that there was more crosstalk between NK cells and T cells in the metastatic group compared to the primary group. It mainly occurs between HLA-1 class molecules (HLA-A, HLA-B, HLA-C) and CD8 A/B. Additionally, in the Ro/e results, we also found that monocytes were significantly enriched in the metastatic group. Metabolic pathway analysis revealed that monocytes in the metastatic group had higher scores in the pentose phosphate pathway, suggesting that metabolic reprogramming might occur in the monocytes of the metastatic samples. In summary, the results mentioned above demonstrate that Panmim is helpful for scientists to explore cancer metastasis in depth.

## Discussion

Metastasis is one of the leading causes of death in cancer patients, involving numerous complex biological mechanisms. During the process of cancer metastasis, there is a close interaction between tumor cells and their surrounding microenvironment. Therefore, an in-depth study of the tumor immune microenvironment is extremely important for revealing the mechanisms of cancer metastasis [43]. The advancement of high-throughput sequencing technology has enabled us to obtain abundant single-cell transcriptomic data. However, this data is often isolated and disorganized. To address this issue, we manually organized the data and adopted a standardized processing workflow for analysis, which ultimately led to the successful construction of Panmim. Panmim contains a total of 90 single-cell transcriptomic datasets related to cancer metastasis. Among these datasets, 52 have a research type of ‘Comparison’ which includes both primary cancer samples and metastatic cancer samples. Additionally, there are 38 datasets with a research type of ‘Single’ which contains only metastatic cancer samples.

Currently, our database has some shortcomings. Firstly, the current data volume is limited, mainly including single-cell transcriptomic data. To expand the coverage of the database, we plan to introduce spatial transcriptomic data, bulk RNA-seq data, and genomic data. Secondly, incorrect annotations of cell types may affect the accuracy of analysis results. In response to this issue, we look forward to developing more precise cell annotation algorithms in the future. Additionally, detailed annotation of cell subpopulations is crucial for revealing the mechanisms of cancer metastasis, so we will increase this part of the work in subsequent studies. To maintain the timeliness and accuracy of the database, we plan to conduct a comprehensive review of the data sources every six months and promptly incorporate new datasets. We firmly believe that Panmim has the potential to become a valuable resource for studying the mechanisms of cancer metastasis.

## Supplementary Information

Additional file 1: Table S1. Detailed information of each dataset in the Panmim database, including the types of primary cancers, metastatic sites, research types, and data sources

## Data Availability

Panmim is freely available online at http://www.gdwk-bioinfo.com/pan_metastasis/home, and there is no login requirement.

## References

[1] Gerstberger S, Jiang Q, Ganesh K. Metastasis. Cell. 2023;186:1564–79. 37059065 10.1016/j.cell.2023.03.003 PMC10511214

[2] Hanahan D, Weinberg RA. Hallmarks of cancer: the next generation. Cell. 2011;144:646–74. 21376230 10.1016/j.cell.2011.02.013

[3] Ye X, Weinberg RA. Epithelial–mesenchymal plasticity: a central regulator of cancer progression. Trends Cell Biol. 2015;25:675–86. 26437589 10.1016/j.tcb.2015.07.012 PMC4628843

[4] Lawson DA, Kessenbrock K, Davis RT, Pervolarakis N, Werb Z. Tumour heterogeneity and metastasis at single-cell resolution. Nat Cell Biol. 2018;20:1349–60. 30482943 10.1038/s41556-018-0236-7 PMC6477686

[5] Tasdogan A, Faubert B, Ramesh V, Ubellacker JM, Shen B, Solmonson A, Murphy MM, Gu Z, Gu W, Martin M. Metabolic heterogeneity confers differences in melanoma metastatic potential. Nature. 2020;577:115–20. 31853067 10.1038/s41586-019-1847-2 PMC6930341

[6] Maquart F-X, Pasco S, Ramont L, Hornebeck W, Monboisse J-C. An introduction to matrikines: extracellular matrix-derived peptides which regulate cell activity: implication in tumor invasion. Crit Rev Oncol Hematol. 2004;49:199–202. 15036260 10.1016/j.critrevonc.2003.06.007

[7] Mlecnik B, Bindea G, Kirilovsky A, Angell HK, Obenauf AC, Tosolini M, Church SE, Maby P, Vasaturo A, Angelova M. The tumor microenvironment and Immunoscore are critical determinants of dissemination to distant metastasis. Sci Transl Med. 2016;8:327–326. 10.1126/scitranslmed.aad6352 26912905

[8] Steeg PS. Tumor metastasis: mechanistic insights and clinical challenges. Nat Med. 2006;12:895–904. 16892035 10.1038/nm1469

[9] Gwee YX, Chia DKA, So J, Ceelen W, Yong WP, Tan P, Ong CA, Sundar R. Integration of genomic biology into therapeutic strategies of gastric cancer peritoneal metastasis. J Clin Oncol. 2022;40:2830. 35649219 10.1200/JCO.21.02745 PMC9390822

[10] Li C, Sun Y-D, Yu G-Y, Cui J-R, Lou Z, Zhang H, Huang Y, Bai C-G, Deng L-L, Liu P. Integrated omics of metastatic colorectal cancer. Cancer Cell. 2020;38(734–747): e739. 10.1016/j.ccell.2020.08.002 32888432

[11] Mi S, Lin M, Brouwer-Visser J, Heim J, Smotkin D, Hebert T, Gunter MJ, Goldberg GL, Zheng D, Huang GS. RNA-seq identification of RACGAP1 as a metastatic driver in uterine carcinosarcoma. Clin Cancer Res. 2016;22:4676–86. 27121792 10.1158/1078-0432.CCR-15-2116

[12] Sun D, Wang J, Han Y, Dong X, Ge J, Zheng R, Shi X, Wang B, Li Z, Ren P, et al. TISCH: a comprehensive web resource enabling interactive single-cell transcriptome visualization of tumor microenvironment. Nucleic Acids Res. 2021;49:D1420–30. 33179754 10.1093/nar/gkaa1020 PMC7778907

[13] Zhao T, Lyu S, Lu G, Juan L, Zeng X, Wei Z, Hao J, Peng J. SC2disease: a manually curated database of single-cell transcriptome for human diseases. Nucleic Acids Res. 2021;49:D1413–9. 33010177 10.1093/nar/gkaa838 PMC7778914

[14] Zeng J, Zhang Y, Shang Y, Mai J, Shi S, Lu M, Bu C, Zhang Z, Zhang Z, Li Y, et al. CancerSCEM: a database of single-cell expression map across various human cancers. Nucleic Acids Res. 2022;50:D1147–55. 34643725 10.1093/nar/gkab905 PMC8728207

[15] Wang Y, Luo J, Jiao S, Xie X, Wang T, Liu J, Shang X, Peng J. STExplore: an integrated online platform for comprehensive analysis and visualization of spatial transcriptomics data. Small Methods. 2025. 10.1002/smtd.202401272. 40045664 10.1002/smtd.202401272

[16] Han Y, Wang D, Peng L, Huang T, He X, Wang J, Ou C. Single-cell sequencing: a promising approach for uncovering the mechanisms of tumor metastasis. J Hematol Oncol. 2022;15:59. 35549970 10.1186/s13045-022-01280-w PMC9096771

[17] Lei Y, Tang R, Xu J, Wang W, Zhang B, Liu J, Yu X, Shi S. Applications of single-cell sequencing in cancer research: progress and perspectives. J Hematol Oncol. 2021;14:91. 34108022 10.1186/s13045-021-01105-2 PMC8190846

[18] Wang R, Song S, Qin J, Yoshimura K, Peng F, Chu Y, Li Y, Fan Y, Jin J, Dang M. Evolution of immune and stromal cell states and ecotypes during gastric adenocarcinoma progression. Cancer Cell. 2023;41(1407–1426): e1409. 10.1016/j.ccell.2023.06.005 PMC10528152 37419119

[19] Wang X, Zhou Y, Wu Z, Xie C, Xu W, Zhou Q, Yang D, Zhu D, Wang M-W, Wang L. Single-cell transcriptomics reveals the role of antigen presentation in liver metastatic breast cancer. Iscience. 2024. 10.1016/j.isci.2024.108896. 38318373 10.1016/j.isci.2024.108896 PMC10839686

[20] Sun L, Kienzler JC, Reynoso JG, Lee A, Shiuan E, Li S, Kim J, Ding L, Monteleone AJ, Owens GC. Immune checkpoint blockade induces distinct alterations in the microenvironments of primary and metastatic brain tumors. J Clin Investig. 2023;133: e169314. 37655659 10.1172/JCI169314 PMC10471177

[21] Clough E, Barrett T. The gene expression omnibus database. Statist Genom Methods Prot. 2016;1418:93–110. 10.1007/978-1-4939-3578-9_5 PMC4944384 27008011

[22] Parkinson H, Kapushesky M, Shojatalab M, Abeygunawardena N, Coulson R, Farne A, Holloway E, Kolesnykov N, Lilja P, Lukk M. ArrayExpress—a public database of microarray experiments and gene expression profiles. Nucleic Acids Res. 2007;35:D747–50. 17132828 10.1093/nar/gkl995 PMC1716725

[23] Database resources of the national genomics data center. China national center for bioinformation in 2022. Nucleic Acids Res. 2022;50:D27–38. 34718731 10.1093/nar/gkab951 PMC8728233

[24] Liu Y, Li Z, Lu J, Zhao M, Qu H. CMGene: a literature-based database and knowledge resource for cancer metastasis genes. J Genet Genomics. 2017;44:277–9. 28527662 10.1016/j.jgg.2017.04.006

[25] Zhang S, He X, Zhang R, Deng W. LncR2metasta: a manually curated database for experimentally supported lncRNAs during various cancer metastatic events. Brief Bioinform. 2021;22:bbaa178. 32766766 10.1093/bib/bbaa178

[26] Zheng G, Ma Y, Zou Y, Yin A, Li W, Dong D. HCMDB: the human cancer metastasis database. Nucleic Acids Res. 2018;46:D950–5. 29088455 10.1093/nar/gkx1008 PMC5753185

[27] Wu S, Zhang J, Wang Y, Qin X, Zhang Z, Lu Z, Kim P, Zhou X, Huang L. metsDB: a knowledgebase of cancer metastasis at bulk, single-cell and spatial levels. Nucleic Acids Res. 2025;53:D1427–34. 39436035 10.1093/nar/gkae916 PMC11701579

[28] Hao Y, Hao S, Andersen-Nissen E, Mauck WM, Zheng S, Butler A, Lee MJ, Wilk AJ, Darby C, Zager M. Integrated analysis of multimodal single-cell data. Cell. 2021;184(3573–3587): e3529. 10.1016/j.cell.2021.04.048 PMC8238499 34062119

[29] McGinnis CS, Murrow LM, Gartner ZJ. DoubletFinder: doublet detection in single-cell RNA sequencing data using artificial nearest neighbors. Cell Syst. 2019;8(329–337): e324. 10.1016/j.cels.2019.03.003 PMC6853612 30954475

[30] Korsunsky I, Millard N, Fan J, Slowikowski K, Zhang F, Wei K, Baglaenko Y, Brenner M, Loh PR, Raychaudhuri S. Fast, sensitive and accurate integration of single-cell data with Harmony. Nat Methods. 2019;16:1289–96. 31740819 10.1038/s41592-019-0619-0 PMC6884693

[31] Hu C, Li T, Xu Y, Zhang X, Li F, Bai J, Chen J, Jiang W, Yang K, Ou Q. Cell Marker 2.0: an updated database of manually curated cell markers in human/mouse and web tools based on scRNA-seq data. Nucleic Acids Res. 2023;51:D870–6. 36300619 10.1093/nar/gkac947 PMC9825416

[32] Ianevski A, Giri AK, Aittokallio T. Fully-automated and ultra-fast cell-type identification using specific marker combinations from single-cell transcriptomic data. Nat Commun. 2022;13:1246. 35273156 10.1038/s41467-022-28803-w PMC8913782

[33] Love MI, Huber W, Anders S. Moderated estimation of fold change and dispersion for RNA-seq data with DESeq2. Genome Biol. 2014;15:1–21. 10.1186/s13059-014-0550-8 PMC4302049 25516281

[34] Robinson MD, McCarthy DJ, Smyth GK. edgeR: a Bioconductor package for differential expression analysis of digital gene expression data. Bioinformatics. 2010;26:139–40. 19910308 10.1093/bioinformatics/btp616 PMC2796818

[35] Wu T, Hu E, Xu S, Chen M, Guo P, Dai Z, Feng T, Zhou L, Tang W, Zhan L. clusterProfiler 4.0: a universal enrichment tool for interpreting omics data. Innovation. 2021;2:100141. 34557778 10.1016/j.xinn.2021.100141 PMC8454663

[36] Wu Y, Yang S, Ma J, Chen Z, Song G, Rao D, Cheng Y, Huang S, Liu Y, Jiang S. Spatiotemporal immune landscape of colorectal cancer liver metastasis at single-cell level. Cancer Discov. 2022;12:134–53. 34417225 10.1158/2159-8290.CD-21-0316

[37] Zhang L, Yu X, Zheng L, Zhang Y, Li Y, Fang Q, Gao R, Kang B, Zhang Q, Huang JY. Lineage tracking reveals dynamic relationships of T cells in colorectal cancer. Nature. 2018;564:268–72. 30479382 10.1038/s41586-018-0694-x

[38] Jin S, Guerrero-Juarez CF, Zhang L, Chang I, Ramos R, Kuan C-H, Myung P, Plikus MV, Nie Q. Inference and analysis of cell-cell communication using Cell Chat. Nat Commun. 2021;12:1088. 33597522 10.1038/s41467-021-21246-9 PMC7889871

[39] Bergers G, Fendt S-M. The metabolism of cancer cells during metastasis. Nat Rev Cancer. 2021;21:162–80. 33462499 10.1038/s41568-020-00320-2 PMC8733955

[40] Cheng PSW, Zaccaria M, Biffi G. Functional heterogeneity of fibroblasts in primary tumors and metastases. Trends Cancer. 2025;11:135–53. 39674792 10.1016/j.trecan.2024.11.005

[41] Guo W, Zhou B, Dou L, Guo L, Li Y, Qin J, Wang Z, Huai Q, Xue X, Li Y, et al. Single-cell RNA sequencing and spatial transcriptomics of esophageal squamous cell carcinoma with lymph node metastases. Exp Mol Med. 2025;57:59–71. 39741182 10.1038/s12276-024-01369-x PMC11799171

[42] McGinnis CS, Miao Z, Superville D, Yao W, Goga A, Reticker-Flynn NE, Winkler J, Satpathy AT. The temporal progression of lung immune remodeling during breast cancer metastasis. Cancer Cell. 2024. 10.1016/j.ccell.2024.05.004. 38821060 10.1016/j.ccell.2024.05.004 PMC11255555

[43] El-Tanani M, Rabbani SA, Babiker R, Rangraze I, Kapre S, Palakurthi SS, Alnuqaydan AM, Aljabali AA, Rizzo M, El-Tanani Y. Unraveling the tumor microenvironment: insights into cancer metastasis and therapeutic strategies. Cancer Letters. 2024;591: 216894. 38626856 10.1016/j.canlet.2024.216894

